# Effect of Daily Intake of *Lactobacillus casei* on Microbial Diversity and Dynamics in a Healthy Pediatric Population

**DOI:** 10.1007/s00284-019-01713-9

**Published:** 2019-06-11

**Authors:** Sofia el Manouni el Hassani, Nanne K. H. de Boer, Fenna M. Jansen, Marc. A. Benninga, Andries E. Budding, Tim G. J. de Meij

**Affiliations:** 1 Department of Pediatric Gastroenterology, Amsterdam UMC, Emma Children’s Hospital, University of Amsterdam, Amsterdam, The Netherlands; 2 Department of Pediatric Gastroenterology, Amsterdam UMC, Emma Children’s Hospital, Vrije Universiteit, Amsterdam, The Netherlands; 3 Department of Gastroenterology and Hepatology, Amsterdam UMC, Vrije Universiteit Amsterdam, AG&M Research Institute, Amsterdam, The Netherlands; 4 Department of Medical Microbiology and Infection Control, Amsterdam UMC, Vrije Universiteit, Amsterdam, The Netherlands

## Abstract

Emerging evidence exists that an altered gut microbiota is a key factor in the pathophysiology of a variety of diseases. Consequently, microbiota-targeted interventions, including administration of probiotics, have increasingly been evaluated. Mechanisms on how probiotics contribute to homeostasis or reverse (effects of) dysbiosis remain yet to be elucidated. In the current study, we assessed the effects of daily Lactobacillus casei strain Shirota (LcS) ingestion in healthy children aged from 12–18 years on gut microbiota compositional diversity and stability. Results were compared to healthy children without LcS exposure. For a period of 6 weeks, fecal samples were collected weekly by both groups. In total, 18 children were included (6 probiotics; 12 non-probiotics). At 1-week intervals, no differences in diversity and stability were observed in children exposed to LcS versus controls. LcS ingestion by healthy children does not result in a more diverse and stable gut microbiota composition. Large double-blind placebo-controlled randomized clinical trials in children should be performed to gain more insight on potential beneficial health consequences.

## Introduction

In the past decade, evidence has emerged on the associations between the microbiota and the pathophysiology of a variety of diseases, such as inflammatory bowel disease (IBD) [1], irritable bowel syndrome (IBS) [2], late onset sepsis (LOS) [3], and necrotizing enterocolitis (NEC) [4]. Therefore, the gut microbiota can be considered as a potential diagnostic biomarker, but also as a potential therapeutic target. Probiotics have been demonstrated to be effective in preventing the development of antibiotic-associated diarrhea and *Clostridium difficile* infections [5]. In addition, probiotics have been shown to be effective in the prevention of NEC in preterm born infants, although data are conflicting [6]. Over the years, the effectiveness of different probiotic strains has been studied, particularly strains from the genera *Bifidobacterium, Saccharomyces*, and *Lactobacillus* [7]. It has been presumed that health benefits are strain-specific, in which functional properties of one strain cannot be extrapolated to other strains [8]. To date, optimal probiotic strains and concentrations for the prevention and treatment of different diseases have not yet been established. Accumulated evidence is provided that supplemented probiotic strains are not incorporated in an individual’s microbiota and, consequently, possible effects disappear after discontinuation [9].

In daily practice, probiotics are used as a food supplement taken by individuals who are not ill, but hope to retain health by taking probiotic supplements in the form of probiotic yoghurts and dairy beverages [10, 11]. To date, however, studies on the effects of probiotics have largely focused on the ability of probiotics to modulate a diseased state towards a healthy state. Interpretation of effects of probiotics on microbiota and clinical symptoms in diseased populations can be complicated by the concomitant use of medication and the course of disease, both influencing microbiota composition. One of the underlying hypotheses is that probiotics could aid in retaining health by stabilizing the gut microbiota, thereby reducing the risk of microbial shifts towards an undesirable composition. Studies on the effects of probiotics, particularly on microbiota composition and dynamics in healthy state, are very limited. Increased knowledge on the effects of probiotics on gut microbiota diversity and stability in healthy populations could possibly lead to a more scientifically-based approach and targeted administration of probiotics in diseased populations. For example, studies on the effects of probiotics containing *Lactobacillus casei* strain Shirota (LcS) on gut microbiota diversity and stability in healthy state are scarce, while therapeutic effects of LcS have been assessed in patients with antibiotics associated diarrhea and functional constipation in several studies, although underlying mechanisms, optimal dosage and duration of administration of LcS remain unclear [12–14]. The aim of the current study was to evaluate the effect of daily ingestion of a fermented milk product containing LcS on the gut microbiota composition, diversity, and short-term dynamics in a healthy pediatric population, compared to healthy children without probiotic exposure.

## Materials and Methods

### Subjects

The current study was embedded in a study in which gut microbiota composition, diversity, and stability in a population of healthy children was studied [15]. In that study, 63 children aged from 2 to 18 years and visiting primary and secondary schools in the Netherlands collected a fecal sample weekly, for a period of 6 weeks and a follow-up sample after 18 months. It was observed that the microbial stability in children varied per phylum, at both short-term and long-term intervals. For the current study, six healthy volunteers aged 12–18 years, who were recruited for the previous study, were instructed to daily ingest probiotics containing LcS during 6 weeks. Effects on composition, diversity and stability of the gut microbiota were assessed. These children were recruited in the same inclusion period, from the same region and collection and analysis of the samples was performed in similar time intervals as the study in which the current study was embedded [15]. Exclusion criteria were the use of antibiotics, probiotics, or immunomodulating agents within 6 months prior to inclusion, culture-proven infectious gastroenteritis 6 months prior inclusion, history of gastrointestinal surgery (except appendectomy), or a diagnosis of a chronic gastrointestinal disease, including functional constipation, celiac disease, IBS, IBD, or short bowel syndrome, which were similarly for children on probiotics and controls. All participants were asked to complete a questionnaire on the following items: age, length, weight, area of inclusion (i.e., agriculture or urban), mode of delivery, pregnancy duration, neonatal feeding mode (i.e., breastfed or formula fed), duration of breastfeeding (if applicable), antibiotic use in the first year of life and medication use during 6 months prior to inclusion and during the study period.

Microbial results of the six included children on LcS were compared to results of 12 children without probiotics from the original cohort of 63 children. It was chosen to include twelve participants (1:2 case–control) to enlarge population size. Participants were matched based with respect to area of inclusion, antibiotics use in the first year of life, feeding mode in the neonatal period and mode of delivery. Participants of both subgroups were not matched based on age, since the assumption that the influence of age on gut microbiota composition is limited after the age of 8 years is previously demonstrated [15]. This study was approved by the Medical Ethics Committee of VU university Medical Center and informed consent was obtained from all study participants and parents in case of children aged under 16 years.

### Probiotics

The probiotic used was a commercially available fermented milk product containing LcS at a minimum concentration of 6.5 × 10^9^ viable cells per 65 mL bottle. The fermented milk was made out of skimmed milk powder, sugar, glucose, and water. The bottles contained 0.8 g proteins, 12 g carbohydrates, <0.1 g fat, and 10 g sugar, providing 50 kcal of energy. Probiotics were taken daily, during breakfast.

### Sample Collection

Collection and analysis of the fecal samples of both studies was performed in the same period. All study participants were asked to collect a fecal sample (approximately 2 grams) in a sterile container (Stuhlgefäß 10 mL, Frickenhausen, Germany) on a weekly basis for a period of 6 weeks. To increase adherence, subjects were instructed to take the probiotics at a fixed moment of the day and to collect the fecal sample at the end of the week. Fecal samples were stored in the freezer (−20 °C) at home within 1 h after collection. Participants were asked to bring the frozen samples in a cooled condition to the outpatient ward of Amsterdam UMC location VUmc.

### DNA Extraction and Sample Preparation

Samples were processed in line with an earlier conducted study [15]. First, DNA was extracted from fecal samples and one sample of the fermented milk product (200 µl) with the easyMag extraction kit according to the manufacturer’s instructions (Biomérieux, Marcy l’Etoile, France). Approximately 100-400 mg feces was placed in an Eppendorf tube with 200 µl of nucliSens lysis buffer and subsequently vortexed. While shaking for 5 min, tubes were incubated at room temperature. After centrifugation (13,000 rmp; 2 min), 100 µl supernatant was transferred to an easyMag isolation container containing 2 ml nucliSens lysis buffer. This suspension was incubated for 10 min at room temperature, after which 70 µl of magnetic silica beads were added. The easyMag automated DNA isolation machine was used following the “specific A” protocol, eluting DNA in 110 µl buffer. All fecal samples were analyzed by intergenic spacer profiling (IS-pro).

### Data Analysis

#### IS-Pro

Preprocessing was carried out with the IS-Pro proprietary software suite (Is-Diagnostics) and resulted in microbial profiles, as has been done in previous study [15]. Three levels of information were obtained: color of peaks sorts species into the phyla Firmicutes, Actinobacteria, Fusobacteria, and Verrucomicrobia (FAFV), *Bacteriodetes*, and *Proteobacteria*, which are the main phyla present in the human gastrointestinal tract [16]. Length of the 16S-23S rDNA IS-region, displayed by number of nucleotides, can subsequently be used to identify bacteria at species level. Specific peak height, measured in relative fluorescence units, reflect the quantity of PCR product. To further analyze the obtained data, each peak in a profile was considered as an operational taxonomic unit (OTU) and its corresponding intensity as its abundance. Species determination of IS-Pro peaks was done by matching of profiles to a database of IS profiles of known bacterial species.

### Diversity and Stability Analysis

The microbial diversity and stability analyses were performed on the IS-pro data. The Shannon diversity index was used as an indicator for the microbial diversity, and was based on the resulting profiles by conventional statistics. The Shannon diversity index was calculated per phylum and for overall microbial composition (by pooling the phyla FAFV, *Bacteriodetes*, and *Proteobacteria*). Diversity analysis was performed with the Statistical Package for the Social Science (SPSS) version 22.0 (IBM, Armonk, USA). The data were visualized by using Spotfire software package (Tibco, Palo, Alto, CA, USA). The gut microbiota compositional stability was defined as intra-individual resistance to change in relative abundances of species over time, quantified by cosine distance (lower distance value represents higher stability). The cosine distance was expressed as a percentage value, for example, when two fecal samples of one individual collected over time would have identical microbial composition; compositional stability was considered to be 100%. The gut microbiota compositional stability of the participants through time was estimated by comparing all intervals per individual (i.e., for 1 week stability, all 1-week intervals were compared). Sample compositions were compared by calculating cosine distances for log2-transformed data per phylum and for the phyla FAFV, *Bacteriodetes*, and *Proteobacteria* combined, as has been done in previous studies [15, 16].

### Demographics

All statistical analysis were performed using SPSS version 22.0 (IBM, Armonk, USA). Demographic and clinical data were compared by an independent *t* test, Mann–Whitney U test or Chi Square test, where considered appropriate. Results were considered significant at a *P* value <0.05.

## Results

### Participants

In total, eighteen participants were included; six children who ingested probiotics and twelve children without probiotics intake. Characteristics of both study groups are depicted in Table 1. Notably, there was no medication use during the study period in both groups. The probiotics group consisted of children with a median age of 13 years, whereas the children in the non-probiotics group were younger (*P* value = 0.03). Consequently, a statistically significant difference was found between both groups in Body Mass Index (BMI); median BMI was higher in the probiotics group compared to the non-probiotics group (*P* value 0.05).

**Table 1:** Subject characteristics

	Probiotics group	Non-probiotics group	*P* value
Study number	6	12	
Gender male (%)	33	33	1
Age, years (median [IQR])	13 [11–17]	9 [8–12]	0.03
BMI (median[IQR])	19 [17–21]	15 [14–19]	0.05
Area of inclusion (*n*)			0.29
Agriculture	0	2	
Urban	6	10	
Mode of delivery (*n*)			0.29
Vaginal	6	10	
Cesarean delivery	0	2	
Pregnancy duration (*n*)			0.6
<37 weeks	0	1	
37–40 weeks	2	2	
>40 weeks	4	9	
Neonatal feeding (*n*)			0.47
Exclusively formula fed	0	1	
Breast milk fed	6	11	
Duration of breastfeeding (*n*)			0.46
<3 months	0	2	
3–6 months	4	4	
>6 months	2	5	
Antibiotic use first year of life (*n*)	1	1	NA
Medication at time of sampling (*n*)	0	0	NA

*BMI* body mass index, *NA* not applicable, *IQR* interquartile range

### *Lactobacillus casei* Strain Shirota Abundance

In the probiotics group, the administered probiotic strain LcS was not detectable in any of the fecal samples analyzed at all predefined time points. Analysis of the fermented milk product by the same IS-pro technique showed that the fermented milk indeed contained the bacterial strain LcS.

### Diversity and Stability

**Fig. 1:**
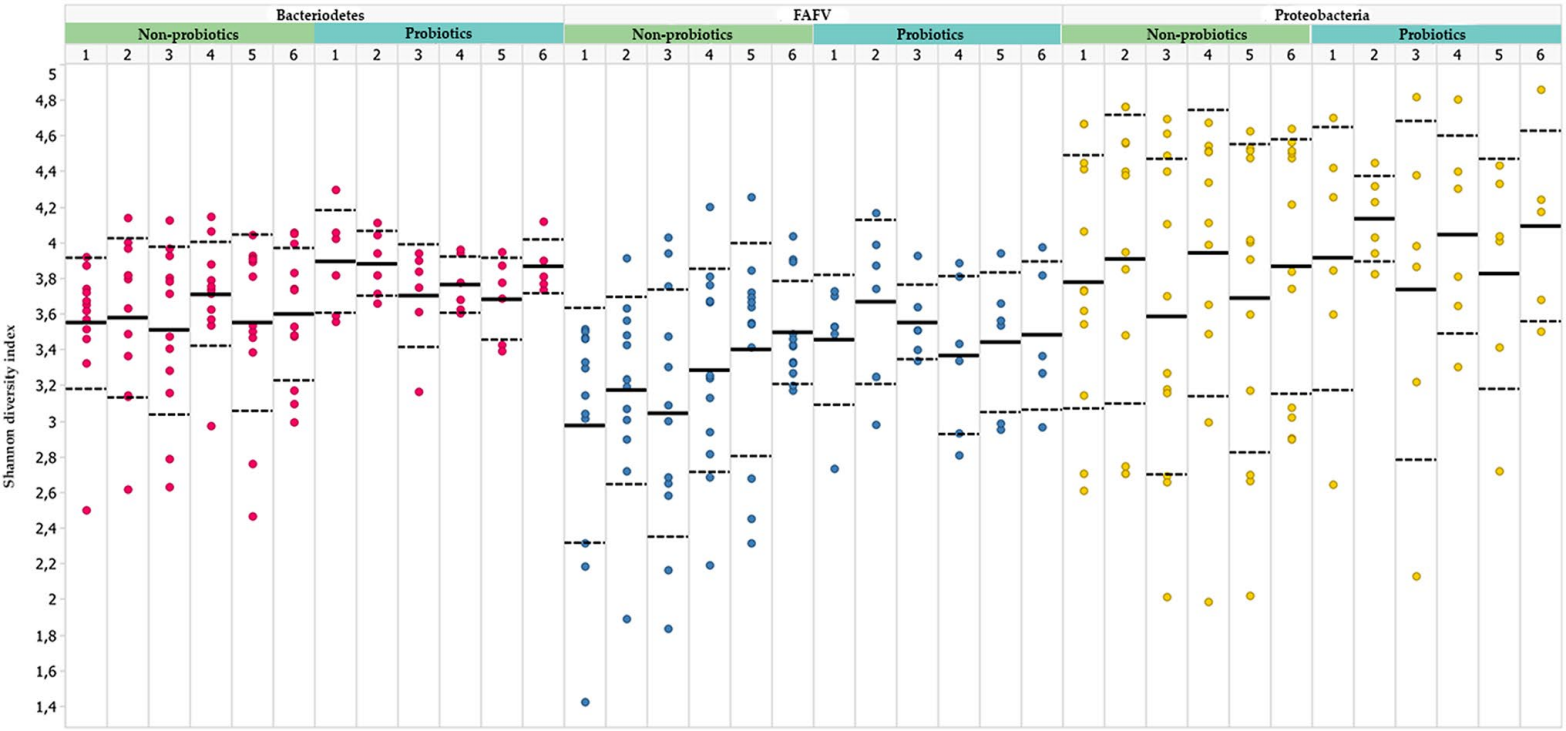
Shannon diversity indices at different time points (t 1–6 correspond with sampling number in weeks), for the phyla *Bacteriodetes* (red), FAFV (blue), *Proteobacteria* (yellow). Dots represent the Shannon diversity index of each subject. No difference was observed in the probiotics group compared to the non-probiotics group at six predefined time points (Color figure online)

No difference was observed in Shannon diversity index in the probiotics group compared to the non-probiotics group at six predefined time points (week 1–6). This observation accounted for all different individual phyla, and was also observed when all phyla were combined (Fig. 1). To enlarge sample size per analysis, a post hoc analysis was performed, in which Shannon diversity indices from all six time points were pooled for both study groups. For this post hoc analysis a linear mixed model was applied. This did not result in a statistically significant difference in diversity index between both study groups (*Bacteriodetes P* value = 0.065; FAFV *P* value 0.071; *Proteobacteria P* value = 0.847).

**Fig. 2:**
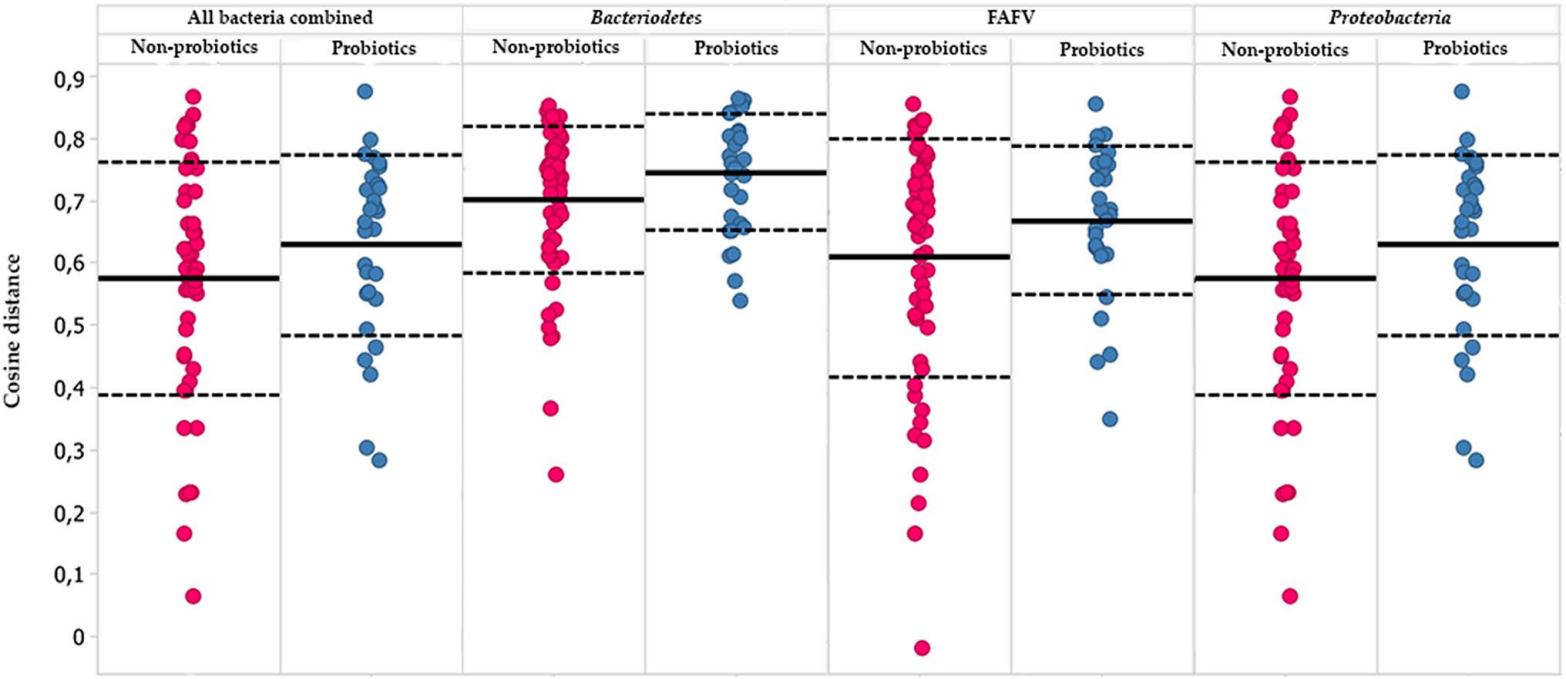
Compositional stability for children with probiotics (blue) and without probiotics (red) exposure, expressed by cosine distance between sequential samples (higher values represent higher stability). Every subject is represented by five dots, since each subject collected six samples with 1-week intervals, therefore allowing for analysis of five 1-week stability measurements (for each subject, week one was compared with week 2, week 2 with week 3, etc.). No difference in compositional stability for all phyla combined and for each different phylum was seen between both study groups (Color figure online)

No difference was observed in 1-week stability between the probiotics and non-probiotics group (Fig. 2) (all bacteria combined *P* value = 0.57, *Bacteriodetes P* value = 0.51, FAFV *P* value = 0.45, *Proteobacteria P* value = 0.48). This was observed both for the total gut microbiota, as for the phyla *Bacteriodetes*, FAFV, and *Proteobacteria* separately.

## Discussion

In the current study, we examined the effect of LcS ingestion on gut microbial stability and diversity in a healthy pediatric population. We demonstrated that daily ingestion of LcS did not result in a higher diversity and stability of the microbiota composition for all phyla, when compared to the control group.

Several studies have assessed the effect of ingestion of LcS in different diseases, such as antibiotic-associated diarrhea [13], functional constipation [14], and NEC [17], with positive results. A study by Wang and colleagues, the effect of daily ingestion of LcS on the microbial compositional diversity and stability in children, was assessed [9]. In this study, it was demonstrated that daily ingestion of LcS resulted in increased levels of *Bifidobacterium* and total *Lactobacillus,* and a decrease of *Enterobacteriacaea, Staphylococcus*, and *Clostridium perfringens*. However, in this study, no control group was included, thereby limiting the ability to draw firm conclusions on the effect of LcS ingestion.

The gut microbiota composition is known to be influenced by numerous factors, such as dietary intake, infections, medication, and area of residence [18, 19]. An undisturbed colonization in early life is related to health in later life, possibly by shaping the immune system [20]. Because low microbial diversity has been linked to various diseased states, manipulation of the microbiota towards a more diverse state has been suggested to be beneficial for the host. However, the evidence for this strategy, besides in the treatment of recurrent *Clostridium difficile* infection, is still weak [21]. In previous studies, a positive correlation was described between the microbial diversity and stability [9, 15]. Recently, a mathematical algorithm was presented which explains the positive correlation between diversity and stability of the gut microbiota [22]. Here, it was demonstrated that higher species diversity leads to higher resilience within small microbiological ecosystems, and therefore results in a more stable system. Interestingly, a study focusing on the role of probiotics in the treatment of diarrhea-dominant IBS demonstrated that the gut microbiota composition in antibiotic-treated patients was more stable during ingestion of probiotics, compared to controls [23]. In healthy state, the phyla *Bacteriodetes* and FAFV have been characterized as phyla with highest diversity, and, possibly as a result, highest stability indices [24]. In the current study, no difference was observed in the diversity indices. However, in the current study, a small sample was used; therefore, additional studies are mandatory to assess the effect of probiotics on the diversity indices in a healthy pediatric population.

In previous studies, it has been demonstrated that the administration of LcS probably affects the gut microbiota composition indirectly, since LcS ingestion influenced abundancies of other strains, rather than an isolated increase in LcS concentrations [9]. Several different mechanisms explaining effects of probiotics have been described, such as immune modulation, the production of lactic acid (consequently reducing local pH), and competitive adhesion or displacement of pathogenic bacteria [25–29]. Studies have reported the advantages on health status of acetic acid produced by intestinal commensals, such as *Bifidobacteria*, which is known to have a bactericidal activity against various pathogenic Gram-negative bacilli [30–32]. Long-term ingestion of LcS has been reported to increase the population levels of indigenous *Bifidobacteria* as well as the fecal concentration of the organic acid levels [33, 34]. In the current study, it has not been demonstrated that LcS ingestion results in a different gut microbiota composition. This could be explained by the sample size, which might not have been sufficient enough to demonstrate potential differences in microbiota composition as has been described earlier. Therefore, not only larger studies are needed, but also additional analyses on bacteria-derived metabolites, which could provide more insight on the mechanisms underlining the effect of LcS on the gut microbiota composition and function.

One of the strengths of the current study is the inclusion of a non-intervention group, allowing for comparison of diversity and stability indices, which are known to fluctuate on short-term intervals, even in healthy state. Another strength was the collection of samples at six time points, allowing for accurate determination of short-term dynamics, in which no significant differences were observed between different time points. In addition, samples were analyzed by means of IS-pro, a molecular microbiota detection technique, which, in contrast to culturing techniques, allows for the identification of the highly complex intestinal microbiota down to species level [15, 16].

This study also has limitations. First, there were no baseline or follow-up measurements prior to or after cessation of LcS ingestion. By including these samples, the effect of daily LcS ingestion on diversity and stability could have been evaluated with more certainty. In addition, by including a sample after probiotics ingestion is ceased, the long-term effect after LcS ingestion could have been evaluated. Another limitation is that participants were not matched based on age. However, in a previous study it was demonstrated in the same population that from the age of 4–18 years, no age-related statistically significant differences in microbial composition were present [15]. Additionally, participants did not record their daily food intake, since detailed understanding of the influence of day-to-day changes in diet on temporal microbial dynamics would need a cohort consisting of at least hundreds of subjects [35, 36]. Therefore, we considered the current series of subjects being too small to perform suitable statistical analysis addressing the influence of diet. The influence of LcS ingestion on gut microbiota composition was assessed by analyzing fecal samples, which is consider to be mainly a reflection of the large intestinal microbiota composition [37–39]. Consequently, little can be said about the effect of LcS ingestion on the small intestinal microbiota composition based on fecal microbiota analyses. However, it has been reported that there is a low bacterial diversity and abundance in the small intestine of healthy individuals [40].

## Conclusions

In the current study, we have explored the effect of ingestion of the probiotic strain LcS on the gut microbiota composition and dynamics in healthy children. There was no higher diversity and stability of the microbiota composition for any phyla after daily LcS ingestion. However, additional studies are needed with a larger cohort in a randomized controlled study design to validate current results.
